# Fractional clearance of urate: validation of measurement in spot-urine samples in healthy subjects and gouty patients

**DOI:** 10.1186/ar4020

**Published:** 2012-08-17

**Authors:** Diluk RW Kannangara, Sheena N Ramasamy, Praveen L Indraratna, Sophie L Stocker, Garry G Graham, Graham Jones, Ian Portek, Kenneth M Williams, Richard O Day

**Affiliations:** 1 Department of Clinical Pharmacology and Toxicology, St Vincent's Hospital, 390 Victoria Street, Darlinghurst, New South Wales 2010, Australia; 2 Department of Pharmacology, University of New South Wales, Wallace Wurth Building, Sydney, New South Wales 2052, Australia; 3 Faculty of Pharmacy, University of Sydney, Pharmacy and Bank Building, Sydney, New South Wales 2006, Australia; 4 Department of Chemical Pathology, St Vincent's Hospital, 390 Victoria Street, Darlinghurst, New South Wales 2010, Australia; 5 Department of Rheumatology, St George Hospital, Gray Street, Kogarah, New South Wales 2217, Australia

## Abstract

**Introduction:**

Hyperuricemia is the greatest risk factor for gout and is caused by an overproduction and/or inefficient renal clearance of urate. The fractional renal clearance of urate (FCU, renal clearance of urate/renal clearance of creatinine) has been proposed as a tool to identify subjects who manifest inefficient clearance of urate. The aim of the present studies was to validate the measurement of FCU by using spot-urine samples as a reliable indicator of the efficiency of the kidney to remove urate and to explore its distribution in healthy subjects and gouty patients.

**Methods:**

Timed (spot, 2-hour, 4-hour, 6-hour, 12-hour, and 24-hour) urine collections were used to derive FCU in 12 healthy subjects. FCUs from spot-urine samples were then determined in 13 healthy subjects twice a day, repeated on 3 nonconsecutive days. The effect of allopurinol, probenecid, and the combination on FCU was explored in 11 healthy subjects. FCU was determined in 36 patients with gout being treated with allopurinol. The distribution of FCU was examined in 118 healthy subjects and compared with that from the 36 patients with gout.

**Results:**

No substantive or statistically significant differences were observed between the FCUs derived from spot and 24-hour urine collections. Coefficients of variation (CVs) were both 28%. No significant variation in the spot FCU was obtained either within or between days, with mean intrasubject CV of 16.4%. FCU increased with probenecid (*P *< 0.05), whereas allopurinol did not change the FCU in healthy or gouty subjects. FCUs of patients with gout were lower than the FCUs of healthy subjects (4.8% versus 6.9%; *P *< 0.0001).

**Conclusions:**

The present studies indicate that the spot-FCU is a convenient, valid, and reliable indicator of the efficiency of the kidney in removing urate from the blood and thus from tissues. Spot-FCU determinations may provide useful correlates in studies investigating molecular mechanisms underpinning the observed range of efficiencies of the kidneys in clearing urate from the blood.

**Trial Registration:**

ACTRN12611000743965

## Introduction

Gout is the most prevalent inflammatory arthritis in men, and its incidence is increasing globally [1]. It is caused by deposition of monosodium urate monohydrate (MSU) crystals in and around joints after long-standing hyperuricemia. Hyperuricemia is defined as a plasma urate concentration greater than 0.42 m*M *and is the most important risk factor for gout, with the risk increasing as plasma urate concentrations increase above this threshold. Hyperuricemia and gout also correlate with risk factors for cardiovascular disease (hypertension, obesity, and diabetes) [2,3].

Urate concentration in the body is governed by dietary purine intake as well as the endogenous synthesis and excretion of urate. Overproduction of urate, as a result of high dietary intake of urate precursors or abnormal enzymatic synthesis, accounts for hyperuricemia in approximately 10% of patients [4]. Approximately two thirds of urate is renally cleared, with the remainder secreted into, and metabolized within, the gastrointestinal tract [5].

It is generally believed that most hyperuricemia in subjects with normal glomerular filtration rates results from a significant inefficiency of the kidneys to clear urate [6,7]. Urate is filtered at the glomerulus, then reabsorbed, and is also secreted by the renal tubules [8]. A variety of urate transporters located in the renal tubules are responsible for the resorption and secretion of urate [8]. The inefficiency of renal clearance in those with normal glomerular filtration rates is considered likely to be the result of impaired tubular secretion, increased tubular resorption, or a combination of both.

A strong genetic influence on renal clearance of urate has been identified, with its heritability being reported as about 87% [9]. To date, however, studies of genetic polymorphisms of identified urate tubular transporter genes [10-12] have been unable to account for the strong genetic influence on the renal clearance of urate.

The fractional clearance of urate (FCU) is the ratio of renal urate clearance to creatinine clearance, thereby expressing urate clearance as a fraction of creatinine clearance. As such, it provides information about the efficiency of the renal tubular mechanisms of urate clearance by correcting for the effect of the glomerular filtration rate, as estimated by the creatinine clearance. It does not measure the amount of urate excreted by the kidney. Thus, it has been proposed that the FCU may be a better correlate than the renal clearance of urate with which to identify subjects whose kidney tubules, inherently, are less efficient at clearing urate from the blood [13].

The FCU has the practical advantage of eliminating the need for the commonly unreliable timed collection of urine needed for direct measures of renal clearance [14-16]. Thus, FCU measurement is potentially more convenient than directly determining the renal clearance of urate that conventionally requires a 24-hour urine collection, in contrast to FCU, which does not require a timed urine collection.

Although the renal clearance of urate decreases with decreasing creatinine clearance down to about 30 ml/min, FCU remains quite stable and increases only marginally [17]. However, below 30 ml/min, the FCU increases disproportionately [17]. Hence, adjusting the renal clearance of urate according to creatinine clearance (that is, calculating the FCU), means that FCU is approximately independent of glomerular kidney function for subjects with reasonable renal function.

FCU can be measured over a period of 24 hours or by spot measurement of plasma and urine concentrations of both urate and creatinine, the spot collection being clearly more convenient. However, the reliability of the spot method is uncertain, perhaps being less stable at various times during 24 hours. Therefore, in the present study, we first examined the reliability of a spot-urine collection to estimate FCU. Second, we explored the effect of probenecid (an uricosuric) and allopurinol (a xanthine-oxidase inhibitor) on FCU values. Third, a spot FCU was investigated in patients with gout before and after treatment with allopurinol. Finally, we examined the distribution of FCU values in healthy volunteers and in subjects with gout.

## Materials and methods

The studies were approved by the St. Vincent's Hospital Human Research Ethics Committee (reference numbers: H05/046, H06/107, 08/SVH/137, 10/093, and 11/SVH/4) and the University of New South Wales Research Ethics Committee, in compliance with the Helsinki Declaration. Written informed consent was obtained from all study participants.

### Patients and study design

The study was conducted in three parts: Part 1 (Validation), Part 2 (Application), and Part 3 (Comparison). Part 1 was broken up into smaller studies, A to D. All subjects were aged between 18 and 80 years.

#### Part 1: Validation

##### Study 1A: Comparison of spot FCU with timed FCU over 24 hours

Healthy, nonsmoking subjects (*n *= 12; seven males) were not permitted alcohol or caffeine for 48 hours and 24 hours, respectively, before the study. Water intake was controlled at 100 ml/h for the 12-hour daytime period of the study. Subject screening included a medical history and a clinical examination followed by routine hematologic and biochemical blood tests (including plasma creatinine and urate concentrations). A spot-urine sample was provided and was immediately analyzed for urate and creatinine concentrations. The subjects began a 24-hour urine collection at 2000 hours. This was divided into a series of consecutive, timed collections of urine (12-hour overnight, followed by a 2-hour, 4-hour, and 6-hour collection). Venous blood samples were taken at the midpoint of the 24-hour period (0800 hours).

##### Study 1B: Variability of spot FCU: morning compared with afternoon

Healthy, nonsmoking subjects (*n *= 13; six males) were not permitted alcohol or caffeine for 48 hours and 24 hours, respectively, before the study. The subjects provided one blood sample (4 ml) in the morning, together with morning and afternoon spot samples of urine on each of the 3 study days with at least 1 day separating each collection. Subjects were asked to void on waking in the morning. Consequently, the spot-urine sample collected in the morning was the second voiding of the bladder for that day. The plasma and urine samples were analyzed for urate and creatinine concentrations. The subjects were encouraged to follow their normal daily routines throughout the study week, although alcohol consumption and vigorous exercise were to be avoided 12 hours and 24 hours, respectively, before the study days.

##### Study 1C: Effect of allopurinol and probenecid on FCU in healthy subjects

Data collected previously as part of an investigation of the interaction between allopurinol and probenecid was used for Study C [18]. In this study, healthy, nonsmoking subjects (*n *= 11, 3 males) were dosed with allopurinol (A; 150 mg, twice daily), probenecid (P; 500 mg, twice daily), or allopurinol plus probenecid (A+P; 150 mg, twice daily, and 500 mg, twice daily, respectively) for up to 7 days to achieve steady state concentrations. This was a three-way, crossover trial with a 14-day washout period before patients entered each arm of the study. Eight serial plasma samples (1, 2, 3, 4, 6, 8, 10, and 12 hours after the morning dose) and 12-hour urine samples were collected at baseline (before treatments) and at Day 8 of treatment. Plasma and urinary creatinine and urate concentrations were determined. Subjects fasted overnight and avoided alcohol and caffeinated drinks for 24 hours before each study day. Complete subject characteristics have been previously reported [18].

##### Study 1D: Effect of allopurinol on FCU in patients with gout

Patients with gout (*n *= 36, 35 males) were recruited. Twenty-two of these patients had not yet begun treatment with allopurinol, whereas the remainder continued the dose of allopurinol being taken at the point of study entry. The dose of allopurinol was titrated incrementally as needed to reduce plasma urate to target concentrations. Blood and spot-urine samples were collected for determination of plasma and urinary creatinine and urate concentrations. This study was registered in the Australian and New Zealand Clinical Trial Registry (ACTRN12611000743965).

#### Part 2: Application: Distribution of FCU in healthy subjects and patients with gout

Eighty-five healthy men recruited to the study provided a single plasma sample and a random spot-urine sample. Plasma and urinary creatinine and urate concentrations were determined from these samples. No restriction was imposed on daily activities or on diet. Demographic features including age, height, weight, and waist circumference were recorded.

All data combined from the subjects in Parts 1 and 2 are termed the SVH subjects (*n *= 154, 118 healthy subjects and 36 subjects with gout). Subjects with data in more than one study (*n *= 3) were considered just once in the grouped SVH subjects cohort. The distribution of FCU in these groups was examined.

#### Part 3: Comparisons: comparison with data from the literature

Data extracted from the literature [7,19-28] were used to calculate and compare FCU values with those from the present study.

### Sample preparation

Plasma samples from healthy volunteers were frozen promptly after collection. Within 2 hours of collection, an aliquot (4 ml) of each urine sample was alkalinized (1.0 *M *NaOH, 3 drops) to prevent formation of less-soluble uric acid and stored (-20°C) for reanalysis, if required.

Urate in plasma and urine was measured by using the uricase method [29], and creatinine, using the Jaffe method by the Pathology Department (Sydpath) of St. Vincent's Hospital Sydney, by using Roche reagents on a Roche Modular Analyser (Roche Diagnostics Australia, Castle Hill, NSW).

### Data and statistical analysis

FCU was calculated as the ratio of urate clearance to creatinine clearance:

(1) $$\text{FCU}=\frac{{\text{U}}_{\text{ur}}\cdot \text{V}}{{\text{P}}_{\text{ur}}}÷\frac{{\text{U}}_{\text{creat}}\cdot \text{V}}{{\text{P}}_{\text{creat}}}$$

The volume terms cancel out, leaving the following formula:

(2) $$\text{FCU}=\frac{{\text{U}}_{\text{ur}}\cdot {\text{P}}_{\text{creat}}}{{\text{P}}_{\text{ur}}\cdot {\text{U}}_{\text{creat}}}$$

Where U and P represent urinary and plasma concentrations, ur is urate, and creat is creatinine. The resultant ratio is expressed as a percentage.

In Study 1A, the FCUs derived from the 24-hour urine-collection measurements were calculated by summation of data from the 2-, 4-, 6-, and 12-hour time periods making up the 24 hours.

Where appropriate, data are presented as the mean and 95% confidence intervals. A repeated measures, one-way analysis of variance (ANOVA) with a Bonferroni *post hoc *analysis was used to examine differences in FCUs (Study 1A, 1C), and a two-way ANOVA with repeated measures was used to compare the interday and intraday FCU (Study 1B). FCUs at various doses of allopurinol in the gouty population (Study 1C) were examined with a one-way ANOVA. An unpaired *t *test was used to examine the differences in FCUs between the healthy subjects and the patients with gout (Part 2). The data were assessed for normality of distribution by using the D'Agostino-Pearson test. FCU values were listed in ascending order and normalized by using the Normdist function in Excel, and the distributions were graphed. Statistical analyses were conducted by using GraphPad (version 5; GraphPad Software, San Diego, CA, USA) and PASW statistics (version 18.0).

## Results

### Part 1: Validation

#### Study 1A: Comparison of spot FCUs with timed FCUs over 24 hours

The mean FCU collected from spot morning urine and plasma samples was similar (7.4%) to the FCUs determined from 24-hour collections (6.9%) (Table 1). The mean FCU of the overnight 12-hour samples was 5.3%. This was considerably lower than that for the timed collection periods during the day. Intersubject coefficients of variation (CV) for spot-urine FCUs and 24-hour urine FCUs were both 28%.

**Table 1:** Fractional clearance of urate (%) in healthy subjects derived from timed urine collections within a 24-hour period

Subject	Spot am	12-hour overnight	2-hour	4-hour	6-hour	24-hour
H01	8.0	5.7	9.9	9.7	7.0	7.1
H02	7.1	3.4	9.0	12.6	7.9	6.6
H03	6.3	3.0	10.6	6.8	6.7	4.8
H04	6.3	5.3	8.5	6.0	8.1	6.4
H05	5.2	3.8	5.2	7.1	6.7	5.2
H06	6.3	5.3	8.5	7.9	7.0	6.4
H07	8.4	6.7	14.3	8.1	7.5	7.8
H08	6.4	7.0	9.0	6.9	7.4	7.3
H09	4.3	2.2	3.0	5.1	6.1	3.8
H10	10.7	11.9	15.8	16.4	12.0	11.1
H11	11.3	2.1	7.1	6.3	9.7	5.8
H12	8.8	7.3	8.5	9.5	7.7	7.9

**Mean**	**7.4**	**5.3**^a^	**9.1^b ^**	**8.5**	**7.8**	**6.9^b^**

**95% CI**	**6.1 to 8.8**	**3.6 to 7.1**	**6.9 to 11.3**	**6.5 to 10.6**	**7.0 to 8.8**	**5.4 to 8.3**

Study 1A, *n *= 12. Urine collection began at 2000 hours with the 12-hour overnight collection. ^a^ Statistically significant difference when compared with other collection periods (*P *< 0.05). **^b^**Statistically significant difference when compared with each other (*P *< 0.05).

#### Study 1B: Variation in spot FCUs in mornings and afternoons

No significant differences were found between morning and afternoon FCUs, although a trend was noted for FCUs to be lower in the morning (*P *= 0.11; 10 subjects showed lower FCU; Table 2). No difference occurred in morning or afternoon FCUs between study days. Individual FCU values were variable, ranging from 4.3% to 16.5%. Two male subjects (V04 and V08) were hyperuricemic (urate concentration, > 0.42 m*M*). An outlier (Table 2, Subject V05 Day 3 afternoon collection) exhibited an unexplained 100% increase in FCU from the morning to the afternoon collection. The plasma and spot-urine samples for this subject were reassayed (postfrozen storage). Less than a 1% variation in urinary urate was measured in thawed samples stored for 3 weeks at -20°C compared with the freshly analyzed samples. With Grubb's test for outliers [30], this value was excluded from the overall analysis. The within-subject CV for FCUs was 16.4% (range, 6% to 23%).

**Table 2:** Fractional clearance of urate (%) in healthy subjects in the morning and afternoon on three separate study days

Subject	Day 1 am	Day 2 am	Day 3 am	Day 1 pm	Day 2 pm	Day 3 pm	Mean
V01	7.8	8.1	8.5	6.8	6.6	7.7	**7.6**
V02	6.1	8.8	6.9	8.8	11.4	9.7	**8.6**
V03	9.7	8.1	4.8	8.5	10.4	7.6	**8.2**
V04	4.2	4.9	4.8	3.4	3.8	4.9	**4.4**
V05	10.6	7.5	8.1	10.0	9.9	16.5	**10.5**
V06	8.8	7.6	6.7	7.8	7.9	7.6	**7.7**
V07	7.3	8.1	11.0	8.5	9.0	10.6	**9.1**
V08	5.7	5.0	6.3	6.7	5.5	8.0	**6.2**
V09	5.9	4.3	4.2	6.1	4.2	6.7	**5.2**
V10	6.4	6.3	7.6	9.5	9.8	9.7	**8.2**
V11	8.9	9.0	9.8	10.2	8.9	10.0	**9.5**
V12	6.7	6.5	9.9	7.9	7.9	8.9	**8.0**
V13	8.7	7.8	6.7	4.8	8.3	7.0	**7.2**

**Mean**	**7.4**	**7.1**	**7.3**	**7.6**	**8.0**	**8.9**	**7.7**

**95% CI**	**6.3 to 8.5**	**6.2 to 8.0**	**6.1 to 8.6**	**6.4 to 8.8**	**6.5 to 9.4**	**7.1 to 10.5**	**6.7 to 8.7**

Study 1B; *n *= 13. Mean age (range) was 35.1 (21 to 73) years.

#### Study 1C: Effect of allopurinol and probenecid on FCU in healthy subjects

At baseline, the mean FCU was 7.9%. Allopurinol did not significantly change the FCU (7.2%) (Table 3). By contrast, FCUs increased approximately threefold when both probenecid and the combination of allopurinol and probenecid were administered. The effect of the combination was not significantly different from the effect of probenecid treatment alone.

**Table 3:** The effect of allopurinol, probenecid, and the combination on fractional clearance of urate in healthy subjects

Subject	FCU (%)			
	
	Baseline	A	P	A + P
H01	4.5	5.7	8.3	14.9
H02	8.1	8.5	25.9	24.1
H03	12.1	7.9	34.0	33.4
H04	4.8	5.4	22.6	26.2
H05	8.2	7.4	22.9	31.7
H06	10.2	10.0	23.1	21.5
H07	5.3	3.9	19.6	23.8
H08	2.5	5.8	16.2	21.4
H09	11.5	10.3	27.4	22.1
H10	12.5	7.5	30.8	30.1
H11	7.5	6.4	17.5	22.9

Mean	7.9	7.2	22.6	24.7

95% CI	5.7 to 10.2	5.8 to 8.5	17.8 to 27.4	21.1 to 28.3

Study 1C; *n *= 11. A, allopurinol; P, probenecid; A + P, the combination. Mean age (range) was 24 (21 to 36) years.

#### Study 1D: Effect of allopurinol on FCUs in patients with gout

**Figure 1:**
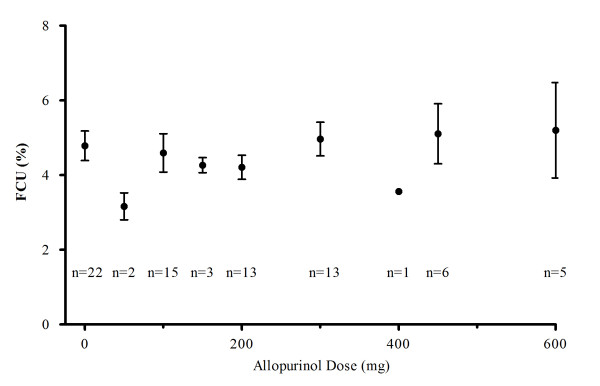
**Fractional clearance of urate CU in gouty patients with incrementally increased doses of allopurinol to achieve target concentrations (Study 1D)**. Mean age (range) was 56 years (17 to 80 years) (*n *= 22). The number of patients at each dose level is indicated on the graph.

The mean FCU in patients with gout before treatment with allopurinol (*n *= 22) was 4.6% (95% CI, 3.8% to 5.4%). Escalation of allopurinol dose did not significantly alter FCUs for these 22 patients (Figure 1). For the total gout cohort (*n *= 36), the mean FCU was 4.8% (95% CI, 4.2% to 5.5%).

### Part 2: Application

**Figure 2:**
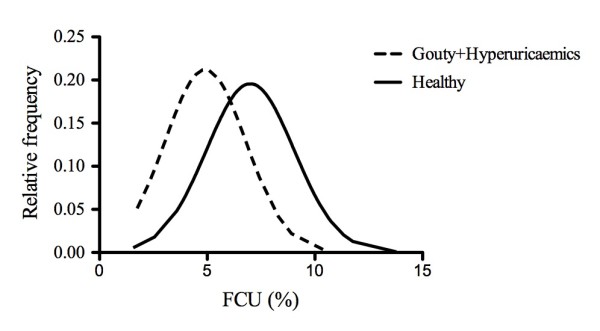
**Distribution of fractional clearance of urate in healthy and gouty subjects in the SVH cohort (Part 2)**.

The mean age of the healthy subjects and gouty patients was 26 years and 56 years, respectively (range, 16 to 80 years). FCUs were normally distributed in both healthy subjects (mean FCU ± standard deviation, 6.9% ± 2.1%) and in gouty patients (mean FCU ± SD, 4.8% ± 1.9%). The mean FCU for all healthy normouricemic subjects (*n *= 110) and hyperuricemic and gouty subjects combined (eight healthy hyperuricemics and 36 gouty patients), was 7.0% ± 2.0% and 4.9% ± 1.9%, respectively (Figure 2). This difference was highly significant, but a large overlap occurred between the two groups. Removing 19 women, all of whom were premenopausal, from the 110 healthy normouricemic patients did not reduce the FCU significantly (from 7.0% ± 2.0% to 6.9% ± 2.1%). Mean ± SD FCUs for women were 7.2% ± 1.5%.

Mean FCU ± SD (95% CI) for the eight healthy hyperuricemics (plasma concentrations > 0.42 m*M*), was significantly lower than for the 110 healthy normouricemics [5.3% ± 1.9%, (3.7 to 6.8) versus 7.0% ± 2.0% (6.6 to 7.4)]. Once again, a large overlap occurred between the two groups.

### Part 3: Comparison

#### Comparison with data from the literature

All FCU values extracted and calculated from the literature are reported in Table 4. In healthy subjects, the mean FCU ranged from 6.5% to 12.8%. Female patients had higher FCU values than did male patients. Subjects with gout had a lower FCU value than did healthy subjects. The FCUs from the SVH cohort were within the ranges of FCUs reported for healthy subjects from the literature. However, comparisons were very difficult because classification of underexcretion, normoexcretion, and overproduction differed markedly between authors (Table 4).

**Table 4:** FCU values in percentage (mean ± SD) extracted and calculated from the literature in healthy and gouty subjects

Reference	Healthy subjects (*n*)	Gouty subjects (*n*)			
		
		Unclassified	Under excretion	Normoexcretion	Overproduction
[19]	-	-	-	5.4 ± 1.0 (5)	6.8 ± 1.6 (8)
[20]	6.9 (36)	-	4.5 (78)	6.0 (46)	-
[21]	-	-	4.3 (26)	-	5.8 (23)
[22]	6.5 ± 2.5 (5)	-	-	-	-
[23]	9.9 ± 1.5 (9)	-	-	-	-
[24]	7.0 ± 1.6 (7)	-	-	5.8 ± 1.3 (10)	8.7 ± 2.7 (6)
[25]	6.9 ± 1.3 (10)	5.1 ± 1.9 (11)	-	-	-
[7]	7.9 ± 3.0 (41) 4.4 ± 1.4 (11 hyperuricemic)	-	-	-	-
[26]	11.1 [female] 8.2 [male] (*n *= 3,224)	-	-	-	-
[27]	8.1 ± 3.2 (51 male) 12.8 ± 2.9 (14 female)	-	-	-	-
[28]	7.6 ± 1.9 (72)	4.6 ± 1.2 (100)	-	-	-

FCU, fractional clearance of urate. Definitions of excretor phenotype as described by respective authors (expressed as percentage per 1.73 m^2 ^body-surface area, where possible). Underexcretion, urinary urate < 826 mg/day/1.73 m^2 ^[20]; urate clearance < 6 ml/min/1.73 m^2 ^[21]. Normoexcretion, urinary urate < 720 mg/day/1.73 m^2 ^[19]; > 830 mg/day/1.73 m^2^) [20]; urate clearance, > 6 ml/min/1.73 m^2 ^[21]; urinary urate < 700 mg/day/1.73 m^2 ^[24]. Overproduction, urinary urate > 720 mg/day/1.73 m^2 ^[19]; > 700 mg/day/1.73 m^2 ^[24].

## Discussion

The renal clearance of urate examines the efficiency of the removal of urate by the kidneys by all mechanisms available to the kidney, whereas the FCU examines the efficiency of the kidneys to remove urate relative to the creatinine clearance. Thus, FCU differs from the rate of renal excretion of urate, which examines the amount of urate removed by the kidneys per unit time. Hence, the term fractional "clearance" of urate (FCU) is perhaps a more accurate descriptor of the ratio of clearance of urate to creatinine than the alternative term sometimes used, the fractional "excretion" of urate [16,28].

The present study established that the FCU could be derived from a spot-urine sample, thereby overcoming some of the uncertainties around the inconvenient and commonly unreliable 24-hour urine collection [15,16], as well as providing a tool to examine more readily the renal tubular urate transport. FCU was unaltered by allopurinol but increased when probenecid was administered, both outcomes being consistent with the accepted mechanism of the action of these drugs (inhibition of urate synthesis by the former and, inhibition of tubular reuptake of urate by urate anion transporter 1 (URAT1) by the latter [31]).

The FCU was marginally higher in the afternoon than in the morning. This difference probably reflects diurnal rhythms in renal function. Vahlensieck *et al*. [32] showed that urinary urate concentrations are highest between 11 a.m. and 5 p.m., and urinary concentrations of creatinine exhibit similar circadian variation [33]. In addition, the low FCU seen at night may be due to the relative dehydration associated with activation of the renin-angiotensin system [13].

The FCU has not been examined widely in studies on urate, although it has been used in heritability studies in twins [9] and correlations on the handling of urate in gouty patients and their relatives [34]. A significant association has been found between a low FCU (≤6.6%) and SLC2A9 variants [11], and thus highlights the potential of the FCU in understanding the function of SNPs in other urate transporters.

In our study, although the FCU was generally greater in women than men, statistical differences were not observed because of the small numbers of healthy female subjects in our studies. This contrasts with the literature, in which the FCU was statistically higher in women [26]. Women are known to have lower plasma concentrations of urate because of the uricosuric effect of estrogen [35], and hence, the incidence of gout is lower in pre- than postmenopausal women [36].

FCU values are available from the literature in both healthy and hyperuricemic/gouty subjects (Table 4), although FCU has generally not been emphasized in such studies. In some cases, only the renal clearances of urate, creatinine, or inulin (marker of glomerular function) were reported, and we have calculated the FCU from these data sets. The present studies also showed that the FCU values were normally distributed, contrasting with a previous study that reported a log normal distribution in healthy subjects [37]. Our results on FCU are consistent with previous data, whereby patients with gout, on average, have a lower FCU than do those without gout. Healthy hyperuricemic patients (that is, without gout) had a lower FCU compared with normouricemic patients, this being in line with the findings of Lang *et al*. [7], whereby the mean FCU of hyperuricemic patients was significantly lower than that of normouricemic patients (4.9% versus 7.0%). It must be noted that gout is largely believed to be related to an inefficient renal clearance of urate, and only in a minority of patients is it due primarily to overproduction of urate. However, some patients with an apparent overproduction, based on the amount of urate secreted in their 24-hr urinary collection output, may also have inefficient renal clearance of urate [28].

A reduced FCU was previously reported in other disease states, including familial juvenile hyperuricemic nephropathy, prerenal azotemia, and idiopathic uric acid nephrolithiasis [24,38-43]. However, a low FCU is not diagnostic of a disease state *per se*, because healthy normouricemic patients with a low FCU are not uncommon, as seen in the present study. Despite a relatively low FCU, many are able to maintain a normal plasma urate, perhaps because of relatively low synthesis of urate. Hyperuricemia was also detected in a small number of healthy subjects with normal FCUs. The clinical implication of a low FCU in an otherwise healthy individual is not known; however, we suggest that these individuals might be more susceptible subsequently to develop hyperuricaemia and gout, especially if their dietary intake of urate precursors is relatively high.

Finally, FCU is not currently proposed for routine clinical use; rather, especially the spot test can be used as a tool in studies exploring the basis for variations in renal handling of urate, including epidemiologic investigations.

## Conclusions

FCU derived from a spot, daytime urine sample is a valid method to provide an estimate of FCU, not different from the FCU estimated by using the traditional 24-hour urine collection. The precision of the spot FCU is comparable to that of other urine-based biochemical tests. Patients with gout have, on average, a lower FCU compared with healthy patients, but considerable overlap exists, and the FCU alone is not predictive of hyperuricemia. A low FCU may be a risk factor for subsequent development of hyperuricemia. Spot FCU may facilitate studies of the renal mechanisms contributing to hyperuricemia.

## Abbreviations

ANOVA: analysis of variance; CI: confidence interval; CV: coefficient of variation; FCU: fractional clearance of urate; P_creat_: plasma creatinine; P_ur_: plasma urate; U_creat_: urinary creatinine; U_ur_: urinary urate.

## Competing interests

The authors declare that they have no competing interests.

## Authors' contributions

All authors contributed equally to the design of the experiments. ROD, GGG, KMW, and IP conceived the studies. DRWK, SNR, PLR, and SLS performed the experiments. GJ gave technical advice. DRWK and SNR drafted the manuscript. All authors read and approved the manuscript for publication.
